# The Effect of Hemoglobin Levels on Mortality in Pediatric Patients with Severe Traumatic Brain Injury

**DOI:** 10.1155/2016/6803860

**Published:** 2016-06-28

**Authors:** Kevin F. Yee, Andrew M. Walker, Elaine Gilfoyle

**Affiliations:** ^1^Department of Anesthesia, Foothills Medical Centre, University of Calgary, 1403 29 Street NW, Calgary, AB, Canada T2N 2T9; ^2^Section of Critical Care, Department of Pediatrics, Faculty of Medicine, University of Calgary, Alberta Children's Hospital, 2888 Shaganappi Trail NW, Calgary, AB, Canada T3B 6A8

## Abstract

*Objective*. There is increasing evidence of adverse outcomes associated with blood transfusions for adult traumatic brain injury patients. However, current evidence suggests that pediatric traumatic brain injury patients may respond to blood transfusions differently on a vascular level. This study examined the influence of blood transfusions and anemia on the outcome of pediatric traumatic brain injury patients.* Design*. A retrospective cohort analysis of severe pediatric traumatic brain injury (TBI) patients was undertaken to investigate the association between blood transfusions and anemia on patient outcomes.* Measurements and Main Results*. One hundred and twenty patients with severe traumatic brain injury were identified and included in the analysis. The median Glasgow Coma Scale (GCS) was 6 and the mean hemoglobin (Hgb) on admission was 115.8 g/L. Forty-three percent of patients (43%) received at least one blood transfusion and the mean hemoglobin before transfusion was 80.1 g/L. Multivariable regression analysis revealed that anemia and the administration of packed red blood cells were not associated with adverse outcomes. Factors that were significantly associated with mortality were presence of abusive head trauma, increasing PRISM score, and low GCS after admission.* Conclusion*. In this single centre retrospective cohort study, there was no association found between anemia, blood transfusions, and hospital mortality in a pediatric traumatic brain injury patient population.

## 1. Background

Traumatic brain injury (TBI) is a leading cause of pediatric morbidity and mortality, accounting for approximately 60,000 hospitalizations and 7,400 deaths per year [1]. Substantial work has been done on anemia and transfusions in both adult and pediatric critically ill patients [2, 3]. Several adult studies have shown an association between anemia, blood transfusions, and poorer outcomes in patients with severe TBI [4, 5].

While the current adult literature is gaining increasing evidence that there may be an adverse effect of transfusion on TBI patients, these results may not be generalizable to pediatric patients. Significant differences exist between pediatric and adult cerebral blood flow (CBF) in both normal and traumatic-injured states [6]. CBF and cerebral metabolic rates are higher in children [6]. In addition, pediatric patients have increased CO_2_ vasoreactivity and have a CBF autoregulation system that is easier to disrupt [6]. The type of blood being used in pediatric patients may also affect the outcome. Lacroix et al. determined that pediatric patients often received leukocyte reduced blood compared to adults who did not [3].

Further, pediatric TBI patients may respond to blood transfusions differently compared to adults. Figaji et al. reported that 79% of their pediatric TBI patients demonstrated an improvement in brain oxygenation after a transfusion [7]. This is in contrast to the 57% of adult TBI patients that had an improvement in brain oxygenation after a blood transfusion [8].

The Transfusion Requirements in Pediatric Intensive Care Unit (TRIPICU) trial demonstrated that adopting a restrictive blood transfusion threshold of a hemoglobin (Hgb) level of 70 g/L in stable critically ill children results in no difference in mortality, but they did not look at TBI patients specifically [3]. Comprehensive evidence-based guidelines have been created to optimize the management of pediatric TBI patients, but there is no consensus on when to transfuse pediatric TBI patients [9]. We performed a retrospective cohort study of pediatric severe TBI patients admitted to the pediatric intensive care unit (PICU) to determine if there is any association between anemia and blood transfusions with mortality in these patients.

## 2. Materials and Methods

Approval from the Conjoint Health Research Ethics Board at the University of Calgary was obtained (Study ID REB13-0095) and informed consent was waived. Patients with a diagnosis of TBI admitted to the Alberta Children's Hospital (ACH) PICU between January 2001 and December 2012 had their charts analyzed by the study investigators. Inclusion criteria were <18 years of age, admission to ACH PICU with diagnosis of TBI or skull fracture, and initial Glasgow Coma Scale (GCS) of ≤8. Exclusion criteria included patient obeying commands within 12 hours of admission, patient death within 12 hours of admission (with injuries likely so severe that blood transfusions would be unlikely to alter their course), nontraumatic etiology to explain decreased level of consciousness (e.g., alcohol or drugs), and concomitant traumatic quadriparesis (unable to assess GCS). These criteria were modelled after a similar study in adult TBI patients [5]. No sampling was done and all patients who met eligibility criteria were included for analysis.

Data was collected on standardized case report forms. Aside from standard demographic information, additional data collected included patient initial hemoglobin and mean hemoglobin for up to the next 7 days after admission; type and severity of the injury characterized by initial vital signs and GCS scores; mechanism of injury; surgical procedures performed; and the presence of signs indicative of raised intracranial pressure (ICP) on CT scans, as reported by a radiologist. At our institution, all patients with severe TBI receive radiologic studies as part of their workup, but not all patients receive invasive ICP monitoring. Presence of intracranial hemorrhage and other injuries was also collected. Recording of patient management data included transfusions in the first 14 days, use of mannitol or hypertonic saline, therapeutic hypothermia, and administration of neuromuscular blockade. Length of hospital and ICU stay along with all-cause mortality within 30 days was used as our primary end-points.

A database containing all previously admitted pediatric patients diagnosed from the desired time period was created. All patients with diagnosis codes of brain injury, traumatic cerebral edema, skull fractures, wounds to head, face, or scalp, and intracranial bleeding admitted to the PICU for more than 24 hours were included in the initial screening.

Presented patient demographic and clinical data are expressed as the mean ± SD or median with interquartile range (IQR) dependent on data normality as assessed using the Shapiro-Wilk test (*p* < 0.05). Given the potential practice change of the publication of the landmark transfusion threshold study in critically ill children by Lacroix et al., we stratified the patients according to whether they were admitted prior to or after 2007 [3]. Significant differences (*p* < 0.05) in continuous demographic and clinical variables between those transfused and not transfused were assessed using the independent samples *t*-test or Mann Whitney *U*-test upon failure of data normality. Significant differences (*p* < 0.05) in categorical variables were assessed using the chi-square test for association or Fisher's exact test.

An exploratory model to predict the probability of mortality was developed using logistic regression. Seven independent variables (age, presence of suspected nonaccidental trauma (NAT), PRISM III score, RBC transnfusion, admission GCS score, admission Hgb, and 7-day mean Hgb) were initially considered as possible covariates. Prior to model construction, a correlation of *ρ* = 0.65 was found between admission Hgb and 7-day mean Hgb using Spearman's rank coefficient. Univariate logistic regression was used to assess the strength of admission and 7-day mean Hgb in predicting the probability of mortality. Nagelkerke *R* ^2^ values were 0.1 and 0.02 for admission and 7-day mean Hgb, respectively, supporting the consideration of admission Hgb as a covariate in our predictive logistic regression model. Additionally, an interaction term of RBC infusion *∗* admission Hgb was also considered as a possible covariate.

The criteria of variable selection for model inclusion followed that of Hosmer Jr. et al. [10]. Briefly, variable selection began with univariate analysis of each independent variable via chi-square analysis for categorical variables (presence of NAT and RBC infusion) and univariate logistic regression for continuous variables. Candidate variables were considered if *p* < 0.2 and as such, all seven covariates were included in an initial predictive model. Results from the initial predictive model of mortality guided the generation of a refined model using only covariates that presented with *p* < 0.05 [10]. The refined, reduced covariate model was compared to the full covariate model using the likelihood ratio chi-square test. All statistical tests were completed using IBM SPSS 20.0 statistics software (IBM, Armonk, NY, USA).

## 3. Results

**Figure 1:**
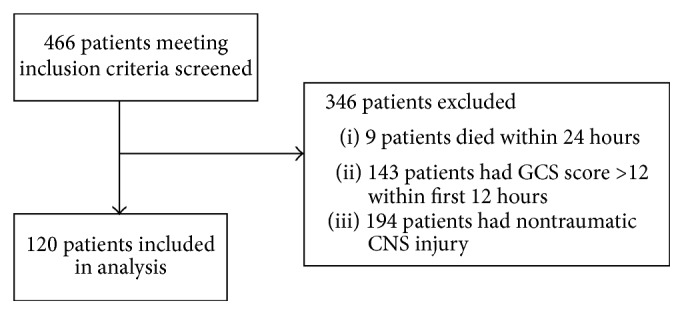
Total number of patients screened and included into the study. TBI: traumatic brain injury. GCS: Glasgow Coma Scale. CNS: central nervous system.

A total of 466 patients with TBI were screened for possible study inclusion (Figure 1). One hundred and twenty met the inclusion criteria. Fifty-three patients (44%) received blood transfusions. Of these patients, the average number of transfusions was 1.67.

Patient demographic and clinical data is summarized in Table 1. Statistically significant differences (*p* < 0.05) between patients transfused and not transfused were noted in age, mortality, PRISM III score, ICU length of stay, hospital length of stay, patients with ICP monitor, initial GCS, surgical procedures performed, presence of raised ICP on imaging, mannitol administration, admission Hgb, nadir Hgb in first 7 days, and mean Hgb over first 7 days. Of the patients who received blood transfusions, 26 patients received invasive ICP monitoring, while 8 patients in the nontransfused group did. One patient was excluded from the length-of-stay calculations as they were transferred to another facility before they were discharged, but their data was used in the main analysis.

The reduced covariate logistic regression model (Table 2) to predict the probability of mortality was statistically significant (*χ* ^2^ = 47.4; *p* < 0.05) and included presence of suspected NAT and PRISM III score as significant (*p* < 0.05) covariates:

(1) Log  odds mortality=−5.294+2.198∗presence  of  suspected  NAT+0.276∗PRISM  III  score.

Odds ratios were 9.01 (95% CI: 2.16–37.68) and 1.32 (95% CI: 1.17–1.48) for presence of suspected NAT and PRISM III score, respectively. The model explained 55.2% of the variance in mortality (Nagelkerke *R* ^2^) and correctly classified 89.1% of patients. Sensitivity and specificity were 50% and 97%, respectively. The likelihood ratio chi-square test was not significant (*χ* ^2^ = 3.70; *p* = 0.594).

## 4. Discussion

In this retrospective cohort study of 120 pediatric patients with severe TBI admitted to our PICU, it was found that hemoglobin at time of admission, administration of blood transfusions, and 7-day average hemoglobin after admission were not associated with adverse outcomes. Significant variables that were associated with increased mortality were the presence of suspected NAT and increasing PRISM score.

It should be noted that there was a significant difference in the patients that were transfused and those that were not. The patients receiving blood transfusions tended to be younger and less well. Despite these differences, there was no clinical outcome difference associated with hemoglobin levels and transfusions.

The goals of TBI management are to prevent secondary insults to the brain after the initial injury, including injury caused by hypoxia and hypotension [9, 11]. Hemoglobin is the major carrier of oxygen in the systemic circulation and there are many conflicting studies examining the relationship between anemia and TBI outcome. Sekhon and colleagues showed that a mean 7-day hemoglobin of less than 90 g/L resulted in increased hospital mortality in severe adult TBI patients [5].

In a large retrospective study of adult TBI patients, Salim and colleagues demonstrated that anemia and correction of it by blood transfusion are associated with increased mortality [4]. Similarly, Warner and colleagues found that adult patients receiving transfusions for moderate anemia after suffering TBI had long-term reduced function at 6 months [12]. In our study, we were not able to record good neurologic outcome data since they were often not documented. Currently, no association between administering blood transfusions and positive outcomes can be made in adult TBI patients and our study supports this assertion in pediatric TBI patients. However, one cannot separate the effects of transfusion with the effects of anemia on outcome in TBI. This is a limitation of the retrospective design of our study, as well as the published adult studies.

Similar to Sekhon and colleagues, we chose to study measured exposure to hemoglobin levels by looking at the initial and average hemoglobin for up to 7 days after admission. A variety of methods have been used to observe hemoglobin levels, but 7 days is thought to capture the occurrence of peak ICP [13]. Like their study, we also captured transfusions as a time-insensitive dichotomous variable that cannot take into context the circumstances surrounding the administration of blood. We recognize that our results may be affected by a survival bias since we did not collect the exact time of death for those patients who died. It is well recognized that, in pediatric critical care, decisions to withdraw life support and the subsequent timing of this withdrawal are heavily affected by the patient's social and cultural backgrounds, so the time of death may not always be based on purely medical circumstances. This makes the link between time of death and severity of illness very difficult in our context [14, 15].

Other limitations of this study include the retrospective nature and the long period of time during which patient information was gathered. During this period of data collection, management strategies may have changed over time, which may have also affected our results. Having said that, our multivariate logistic regression analysis did not show an association between other management strategies and outcomes. All patients reviewed were admitted to a single centre suggesting that our results may not be entirely generalizable to other hospitals. However, the fact that our study found associations between NAT, GCS, and PRISM scores suggests that our study population is similar to other pediatric TBI studies that identified these as factors as well [16–18].

In conclusion, initial admission Hgb and mean Hgb values averaged over seven days after admission to PICU were not found to be strong predictors of mortality in children with severe TBI. No difference in outcome can be demonstrated when patients are transfused. Based on our study, we cannot advocate for deviation from the widely accepted transfusion threshold of 7 g per deciliter in critically ill pediatric patients presenting with TBI [3]. Prospective evaluation of the association among anemia, transfusion, and outcome should be undertaken to further define any possible relationship.

## Figures and Tables

**Table 1:** Demographic information. GCS: Glasgow Coma Scale. Hgb: hemoglobin. PRISM III: pediatric risk of mortality III score. SD: standard deviation. Values represented as “*x* (*y*)” are the median (interquartile range). Values with “*x* ± *y*” are mean ± SD.

Clinical variable	Patients transfused (*n* = 53)	Patients not transfused (*n* = 67)	*p* value
Mean age (years)	3.5 (3.5)	11.3 (10.6)	**<0.005**
Male gender (%)	32 (60)	47 (70)	0.262
Year of admission (%)			
2001–2006	28 (52)	34 (51)	0.821
2007–2012	25 (48)	33 (49)	0.821
Mechanism of injury			
Motor vehicle accident	23 (43)	26 (39)	0.611
Fall	3 (6)	9 (13)	0.159
Assault	10 (19)	10 (15)	0.565
Accidental	17 (32)	22 (33)	0.930
Suspected nonaccidental trauma (%)	12 (23)	14 (21)	0.818
Mortality (%)	13 (25)	7 (10)	**0.040**
PRISM III score	10.0 (9)	5.0 (2)	**<0.005**
Mean ICU length of stay (days)	6.0 (6)	3.0 (2)	**<0.005**
Mean hospital length of stay (days)	15.0 (22)	9.0 (13)	**0.049**
Patients with ICP monitor (%)	26 (49)	8 (12)	**<0.005**
Mean daily ICP (mmHg)			
Day 1	12.0 (8)	17.0 (14)	0.624
Day 2	14.0 (8)	16.0 (12)	0.815
Day 3	16.0 (8)	18.0 (8)	0.477
Day 4	14.0 (8)	15.0 (13)	0.673
Day 5	14.0 (10)	18.0 (53)	0.622
Day 6	18.0 ± 10.8		
Day 7	16.5 ± 4.4		
Admission Glasgow Coma Scale (GCS)	3.0 (4)	6.0 (4)	**0.009**
Surgical procedures performed (%)			
0	27 (51)	55 (82)	**<0.005**
1	10 (19)	7 (10)	0.189
2	16 (30)	5 (7)	**0.001**
Presence of raised ICP on imaging (%)	32 (60)	20 (30)	**0.001**
Presence of intracranial hemorrhage (%)	39 (74)	39 (58)	0.079
Intracranial hypertension medical management (%)			
Mannitol admin	21 (40)	10 (15)	**0.002**
3% NaCl admin	6 (11)	4 (6)	0.292
Hypothermia	8 (15)	9 (13)	0.795
Use of neuromuscular blockade (%)	21 (40)	19 (28)	0.194
Admission Hgb (g/L)	101.6 ± 24.6	126.7 ± 13.9	**<0.005**
Nadir Hgb in first 7 days (g/L)	83.0 (20)	105.0 (18)	**<0.005**
Mean Hgb over first 7 days (g/L)	106.8 (12)	115.8 (14.5)	**<0.005**
Mean number of transfusions	1.67 ± 1.78		

**(a):** 

Variables	β	Standard error	*p* value	Odds ratio	95% CI odds ratio (lower-upper)
NAT suspected	2.434	0.989	0.014	11.40	1.64–79.25
PRISM III score	0.222	0.072	<0.005	1.25	1.08–1.44
Age	0.039	0.094	0.679	1.04	0.87–1.25
Admission GCS	−0.303	0.226	0.179	0.74	0.47–1.15
Initial Hgb	−0.057	0.042	0.174	0.95	0.87–1.03
RBC transfusion	−5.688	5.366	0.289	0.00	0.00–125.17
Initial Hgb *∗* RBC transfusion	0.046	0.045	0.302	1.05	0.96–1.14
Constant	3.094	5.468	0.572	22.06	

*R* ^2^ = 0.586; χ^2^ = 51.1.

**(b):** 

Variables	β	Standard error	*p* value	Odds ratio	95% CI odds ratio (lower-upper)
NAT suspected	2.198	0.730	<0.005	9.011	2.155–37.684
PRISM III score	0.276	0.060	<0.005	1.317	1.171–1.482
Constant	−5.294	0.937	<0.005	0.005	

*R* ^2^ = 0.552; χ^2^ = 47.4.

NAT: nonaccidental trauma; GCS: Glasgow Coma Scale; Hgb: hemoglobin; RBC: red blood cell.
